# Development and validation of programmed cell death related genes in intracranial aneurysms

**DOI:** 10.1186/s12883-026-04794-9

**Published:** 2026-03-11

**Authors:** Ning Gan, Min Ge, Yaxin Wang, Feifan Ma, Huiqing Liang, Yan Zhai, Rongcai Jiang

**Affiliations:** 1 Department of Neurosurgery, State Key Laboratory of Experimental Hematology, Laboratory of Post‐Neuroinjury Neurorepair and Regeneration in Central Nervous System Tianjin & Ministry of Education, Tianjin Medical University General Hospital, Tianjin, 300052 China; 2 Department of Neurosurgery, Baoding No.1 Central Hospital, Baoding, Hebei 071000 China; 3 Department of Neurosurgery, Baoding No.1 Hospital, Baoding, Hebei 071000 China; 4 Department of Neurosurgery, Xuanwu Hospital, Capital Medical University, Beijing, 100003 China

**Keywords:** Intracranial aneurysm, Ferroptosis, Immunogenic cell death, Machine learning, Bioinformatics

## Abstract

Intracranial aneurysm (IA) is a life-threatening condition. Programmed cell death (PCD) is central to aneurysm onset and progression. Nevertheless, a comprehensive analysis of the association between PCD and IA is lacking.We retrieved five IA-dataset including GSE13353, GSE15629, GSE26969, GSE54083, and GSE75436 from GEO database. After integrating these datasets, We calculated 9 cell death pattern scores via ssGSEA and identified ferroptosis and immunogenic cell death(ICD) as the higher relevance in the IA (AUC-ROC > 0.7).We relied on differential gene analysis and took intersections with ICD- and ferroptosis- genes, which was as the IA-PCD genes. Integrating Least absolute shrinkage and selection operator (LASSO) and support vector machine recursive feature elimination (SVM-RFE), identified two key genes, namely NLRP3 and HMOX1*.* In the dataset, the two genes were significantly overexpressed in patients with IA. Immune infiltration analysis showed that *NLRP3* and *HMOX1* could influence the extent of immune cell infiltration.For further validation, we found that the expression levels of NLRP3 and HMOX1 in the serum of IA patients were significantly higher than those in the normal group though Elisa, consistenting with the bioinformatics result. In conclusion, NLRP3 and HMOX1 are associated with IA. These findings provide a basis for further exploring potential molecular driving mechanisms and screening suitable biomarkers.

**Supplementary Information:**

The online version contains supplementary material available at 10.1186/s12883-026-04794-9.

## Introduction

Intracranial aneurysm (IA) is a critical health condition, characterized by localized dilation or abnormal bulging of cerebral arteries, often presenting as cystic aneurysms, affecting approximately 2% to 5% of adults worldwide [1, 2]. Whereas several aneurysms remain stable, some are susceptible to rupture, leading to aneurysmal subarachnoid hemorrhage (aSAH). aSAH has a mortality of approximately 40% [3]. Patients with aSAH often experience reduced quality of life, placing substantial psychological and financial burdens on families and individuals [4]. The pathophysiology of IA is complex and not entirely understood. Contributing factors comprise high shear stress on vessel walls, vascular inflammation, and endothelial dysfunction [5]. Macrophage activation is central to IA formation and rupture by inducing apoptosis through the release of inflammatory factors and matrix metalloproteinases. This necessitates identifying novel biomarkers and exploring the mechanisms underlying IA progression. These advancements will facilitate early IA diagnosis and improve patient prognosis.

Various forms of programmed cell death (PCD), including pyroptosis, ferroptosis, and necroptosis, are implicated in neurological disorders. Several neurological conditions are characterized by abnormalities in the PCD pathway, indicating that the pharmacological modulation of PCD may regulate IA treatment [6]. This study utilized bioinformatics to investigate the role of PCD in IA, aiming to identify key PCD genes, construct diagnostic markers, and validate the findings via Enzyme-Linked ImmunoSorbent Assay in the clinical samples. These findings will provide a theoretical foundation for developing non-surgical treatment approaches.

## Methods

### Identifying IA-associated key PCD patterns

**Fig. 1:**
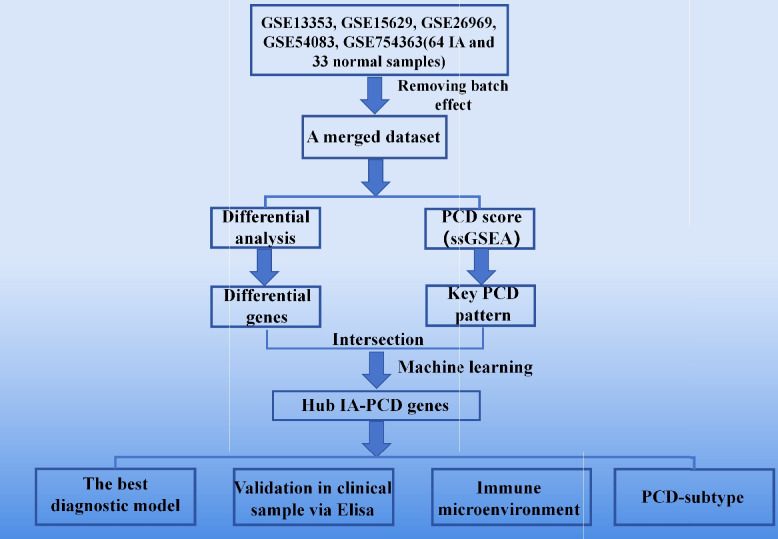
Flowchat

Figure 1 represented the flow chart of this study. Five independent IA datasets [7, 8] including GSE13353, GSE15629, GSE26969, GSE54083, and GSE75436, were downloaded from the Gene Expression Omnibus database (http://www.ncbi.nlm.nih.gov/geo/). The R package “sva” utilizes a Bayesian framework to correct batch effects. Initially, the input data is structured as a matrix, with rows representing genes and columns representing samples. The data is normalized, setting the mean of each gene within each batch to 0 and the variance to 1. Subsequently, a linear model estimates the parameters of the batch effect for each gene. An empirical Bayesian approach is then employed to stabilize these parameter estimates by leveraging information from all genes. Specifically, the mean and variance are calculated for each batch and adjusted using a Bayesian method. Lastly, the data is adjusted for the estimated batch effect parameters. A set of nine PCD pattern-related genes was obtained based on previous studies [9] (Suppl Table 1). After removing batch effects, the datasets were merged. The “GSVA” R package was used to conduct single-sample gene set enrichment analysis on these datasets [10]. Using the wilcox test to compare the differences in PCD mode scores between IA samples and healthy samples (*P* < 0.05), we excluded the mode with no significant difference in score. Receiver operating characteristic (ROC) curves were plotted to assess the diagnostic performance of each pattern. PCD patterns with an area under the ROC curve (AUC) > 0.7 were identified as the key patterns. Finally, all genes associated with the key patterns were combined into the PCD gene set.

### Differential gene analysis

The “limma” R package was used to conduct differential gene analysis on the merged IA dataset. Genes meeting the criteria of |log2FoldChage|> 0.585 and adjusted *p*-value < 0.05 were used to obtain the differentially expressed genes (DEGs). Results were plotted using volcano plots. PCD-related DEGs (PCD-DEGs) were identified by considering the intersection of the PCD gene set and DEGs.

### Optimal diagnostic model

First, Least Absolute Shrinkage and Selection Operator (LASSO) regression and Support Vector Machine-Recursive Feature Elimination (SVM-RFE) via the R packages “glmnet” and “e1071” were used to identify the key PCD-DEGs associated with IA. Second, the dataset was split into training (60%) and validation (40%) cohorts. Third, diagnostic models, including SVM, efficient neural network (ENet), eXtreme Gradient Boosting (Xgboost), and decision tree (DT), were constructed and evaluated in the validation cohort using Matthews correlation coefficient (MCC), accuracy, and precision. Finally, the best-performing diagnostic model was selected.

### Immune infiltration

Immune cell infiltration was assayed using R package “Cibersort”. The Wilcox test was used to compare differences in immune cell proportions between the IA and healthy groups (*P* < 0.05). Spearman correlation analysis was conducted to assess the correlations between key genes and immune cells (Correlation coefficient > 0.3, *P* < 0.05).

### PCD-associated IA

Based on PCD-DEGs, the “Consensus Clustering” R-package was used to cluster aneurysm samples into subtypes. Differences in immune cell infiltration and regulatory pathways among subtypes were compared.

### Validation of hub genes

#### Data collection

This study involved patients with IA admitted to the Department of Neurosurgery at the No.1 Central Hospital of Baoding City between January 2023 and June 2024. The inclusion criteria were as follows: (i) subarachnoid hemorrhage (SAH) secondary to intracranial aneurysm confirmed via cranial computed tomography angiography or digital subtraction angiography. (ii) Hunt-Hess grade between 1 and 4. (iii) After admission, all patients underwent intracranial aneurysm embolization, with no surgical complications and no delayed hydrocephalus The exclusion criteria were as follows: (i) other causes of SAH. (ii) intracranial vascular anomalies and (iii) incomplete clinical data. We also obtained twenty blood samples from healthy people at the health center in the hospital, served as the control group. In previous studies, it was difficult to accurately estimate the sample size due to the difficulty in obtaining the mean and standard deviation of NLRP3 and HO-1 serum in IA patients.We determined the sample size based on 10 Events Per Variable(EPV) methods, where each independent variable corresponds to at least 10 positive samples [11]. The study was approved by the Institutional Ethics Committee of Baoding No.1 Central Hospital (Number: [2023]−153) and written informed consent was obtained from all participants.

#### Enzyme-linked immunosorbent assay

Five milliliters of fasting blood samples were collected from all participants. After natural coagulation at room temperature for 10 to 20 min, the samples were centrifuged at 12,000 × *g* for 15 min at 4 °C. The supernatant was transferred to sterile Eppendorf tubes. Elisa kits were purchased from Sino Biological Ltd Company (Beijing, China) to measure the levels of *HO-1* and *NLRP3.*

#### Statistics

Statistical analysis was conducted using R version 4.2.1. Normally distributed data are presented as mean ± standard deviation, and comparisons between groups were conducted using the t-test. Non-normally distributed data are presented as median (P25, P75), and the Mann–Whitney U-test was used for group comparisons. *P*-value < 0.05 indicated statistical significance.

## Results

### Key cell death patterns

**Fig. 2:**
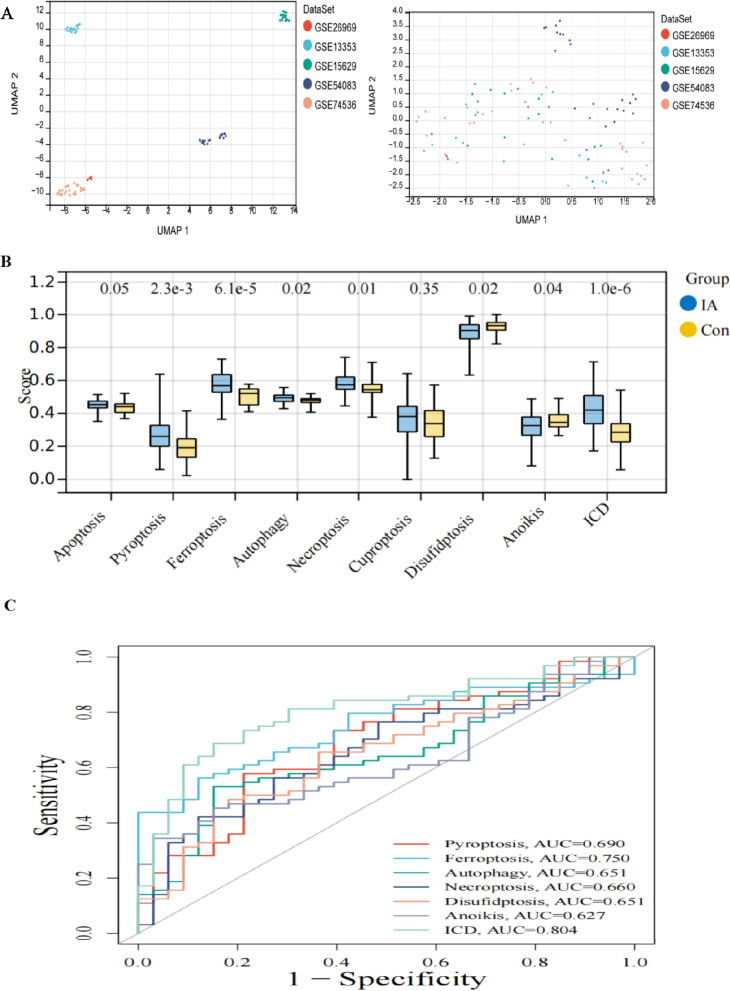
Identification of key cell death patterns in IA (**A**) batch effect (**B**) differential analysis of scores for 9 cell death patterns (**C**) diagnosis of ROC curve area under cell death pattern scores

Five datasets were analyzed after removing batch effects, yielding 64 IA (intracranial aneurysm tissue) and 33 healthy samples(normal superficial temporal artery) (Fig. 2A). Figure 2B suggests that scores for pyroptosis, ferroptosis, autophagy, necroptosis, anoikis, and immunogenic cell death (ICD) were significantly higher in the IA group, compared with the healthy group (*P* < 0.05). However, there was no difference in Apotosis and Cuproptosis scores between the groups. Contrarily, disulfidptosis scores were markedly higher in the healthy group, compared with the IA group. Figure 2C demonstrates that the AUC for ferroptosis and ICD exceeded 0.7. Consequently, ferroptosis and ICD were considered key modes of PCD, comprising 116 genes in the PCD-associated gene set.

### Key PCD-DEGs

**Fig. 3:**
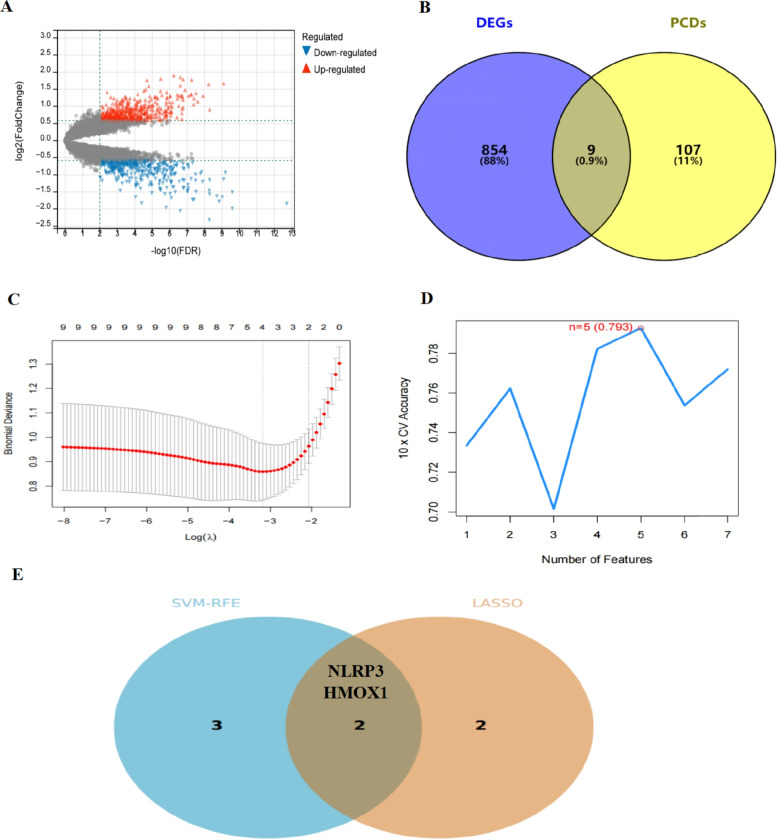
Key PCD identification (**A**) volcano map of IA differential genes (**B**) PCD differential genes (**C**) lasso identification of IA key PCD genes (**D**) SVM-RFE identification of IA key PCD genes (**E**) IA related core PCD genes

A total of 863 DEGs were eligible for screening (Fig. 3A-B). Of these, nine PCD-DEGs were obtained. Next, we used lasso or SVM-RFE algorithms for screening, obtaining four or five feature genes, respectively (Fig. 3C and D). Furthermore, we intersected the results of the two algorithms to get NLRP3 and HMOX1 genes, indicating their significant contributions in different models. Based on this, we speculated that they could play a critical role in IA. In addition, referring to previous studies, the concentration of NLRP3 and HO-1 proteins(HMOX1) could be detected in the serum via Enzyme-linked immunosorbent assay(Elisa) [12, 13]. This indicated that the proteins encoded by NLRP3 and HMOX1 genes could be detected in the serum, providing a basis for validation using blood samples from IA patients.

### Model performance

**Fig. 4:**
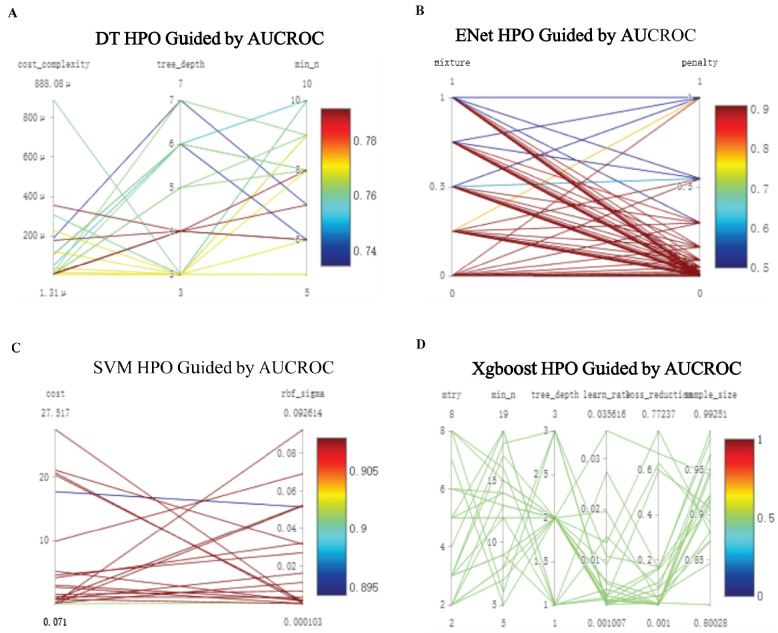
Machine learning model hyperparameter optimization (**A**) decision tree (**B**) elastic network (**C**) support vector machine (**D)** eXtreme gradient boosting

Hyperparameter tuning was conducted for each model, and the results were assessed (Fig. 4A–D). After using the best parameters, four models including SVM, DT, eNet and Xgboost were built and validated in the testdata. As shown in Table 1, SVM and eNet performed better than the other two among these indicators such as Accuracy, MCC, ROC_auc, PR_auc. It is generally believed that the closer the Brier Score is to 0, the higher the accuracy of the model. Therefore, SVM performed better than eNet(Brier score:0.17 vs 0.20),

**Table 1:** The performance in the testdata

Model	Model evaluation				
	Accuracy	MCC	ROC_auc	PR_auc	Brier score
Decision Tree	0.70	0.36	0.65	0.80	0.23
Elastic Network	0.73	0.52	0.85	0.93	0.20
eXtreme Gradient Boosting	0.65	0.00	0.50	0.83	0.25
Support vector machine	0.73	0.52	0.84	0.92	0.17

### Immune infiltration analysis

**Fig. 5:**
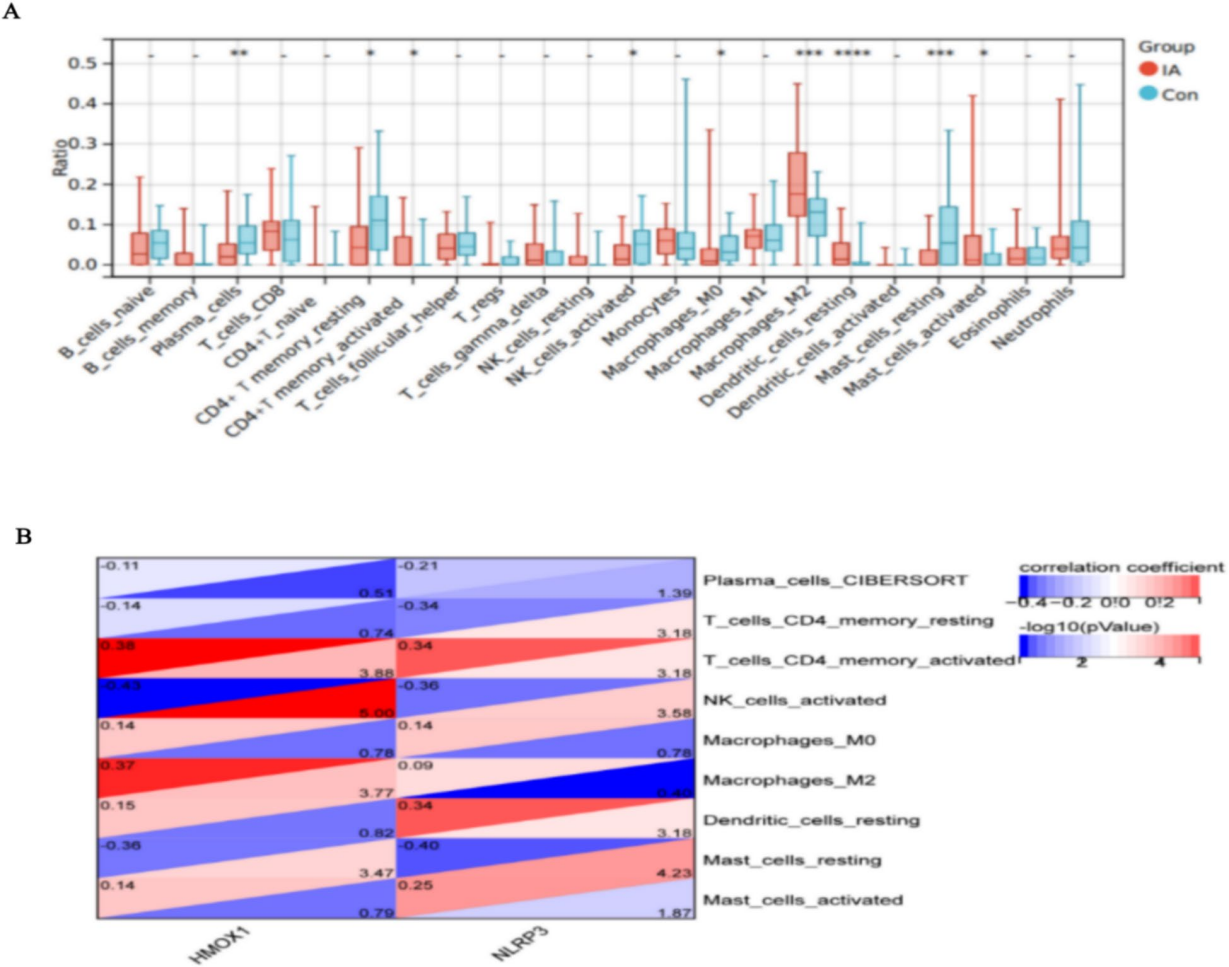
Analysis of immune cell infiltration and its correlation with core PCD genes (**A**) immune infiltration (**B**) correlation analysis

Compared with the healthy group, the levels of activated memory CD4 + T cells (*p* < 0.05), M2 macrophages (*p* < 0.001), resting dendritic cells (*p* < 0.0001), and activated mast cells (*p* < 0.05) were higher in the IA group (Fig. 5A). Contrarily, the levels of plasma cells (*p* < 0.01), resting memory CD4 + T cells (*p* < 0.05), activated natural killer (NK) cells (*p* < 0.05), M0 macrophages (*p* < 0.05), and resting mast cells (*p* < 0.001) were lower in the IA group, compared with the healthy group. As shown in Fig. 5B, spearman correlation analysis indicated that HMOX1 was positively correlated with M2 macrophages (correlation coefficient = 0.37, *p* < 0.01) and CD4 + T memory activated cells (correlation coefficient = 0.38, *p* < 0.01), whereas negatively correlated with resting mast cells (correlation coefficient = −0.32, *p* < 0.01) and actived NK cells(correlation coefficient = −0.43, *P* < 0.01). Similarly, NLRP3 was positively correlated with activated CD4 + T memory activated cells (*r* = 0.34, *p* < 0.01) and, Dentritic resting cells(*r* = 0.34, *p* < 0.01), whereas positively correlated with activated resting mast cells (*r* = −0.40, *p* < 0.05) and actived NK cells (*r* = −0.36, *P* < 0.01).

### Biological characterization of different PCD patterns

**Fig. 6:**
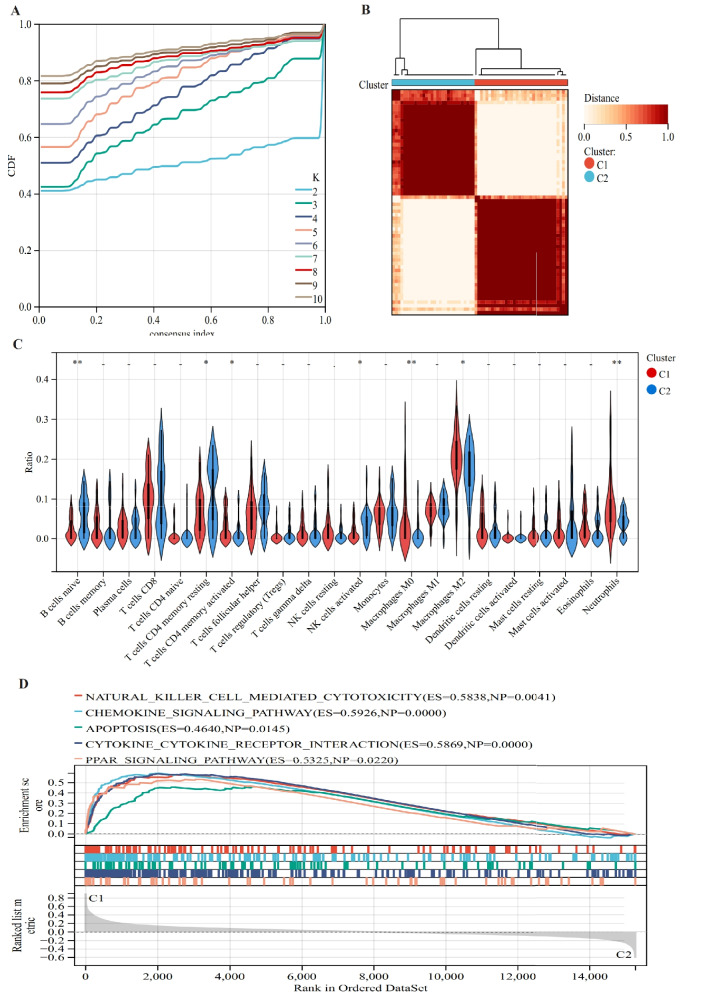
Biological characteristics of PCD subtypes (**A**-**B**) based on 9-PCD differential genotyping (**C**) immune infiltration cell differences between subtypes (**D**) C1 subtype related regulatory pathways

IA samples were divided into two clusters (C1, C2) based on nine PCD-DEGs (Fig. 6A and B). As show in Fig. 6C, immune infiltration analysis suggested that cluster C1 was elevated in M0 (*p* < 0.01) and M2 macrophages (*p* < 0.05). By contrast, cluster C2 was elevated in naïve B cells (*p* < 0.01), resting memory CD4 + T cells (*p* < 0.05), regulatory T cells (*p* < 0.05), activated NK cells (*p* < 0.01), and monocytes (*p* < 0.05). Further, GSEA analysis demonstrated that the C1 subtype was primarily regulated by pathways related to apoptosis, chemokine signaling, and peroxisome proliferator-activated receptor signaling (Fig. 6D).

### Serum *NLRP3* and *HO-1* levels between IA and healthy groups

**Fig. 7:**
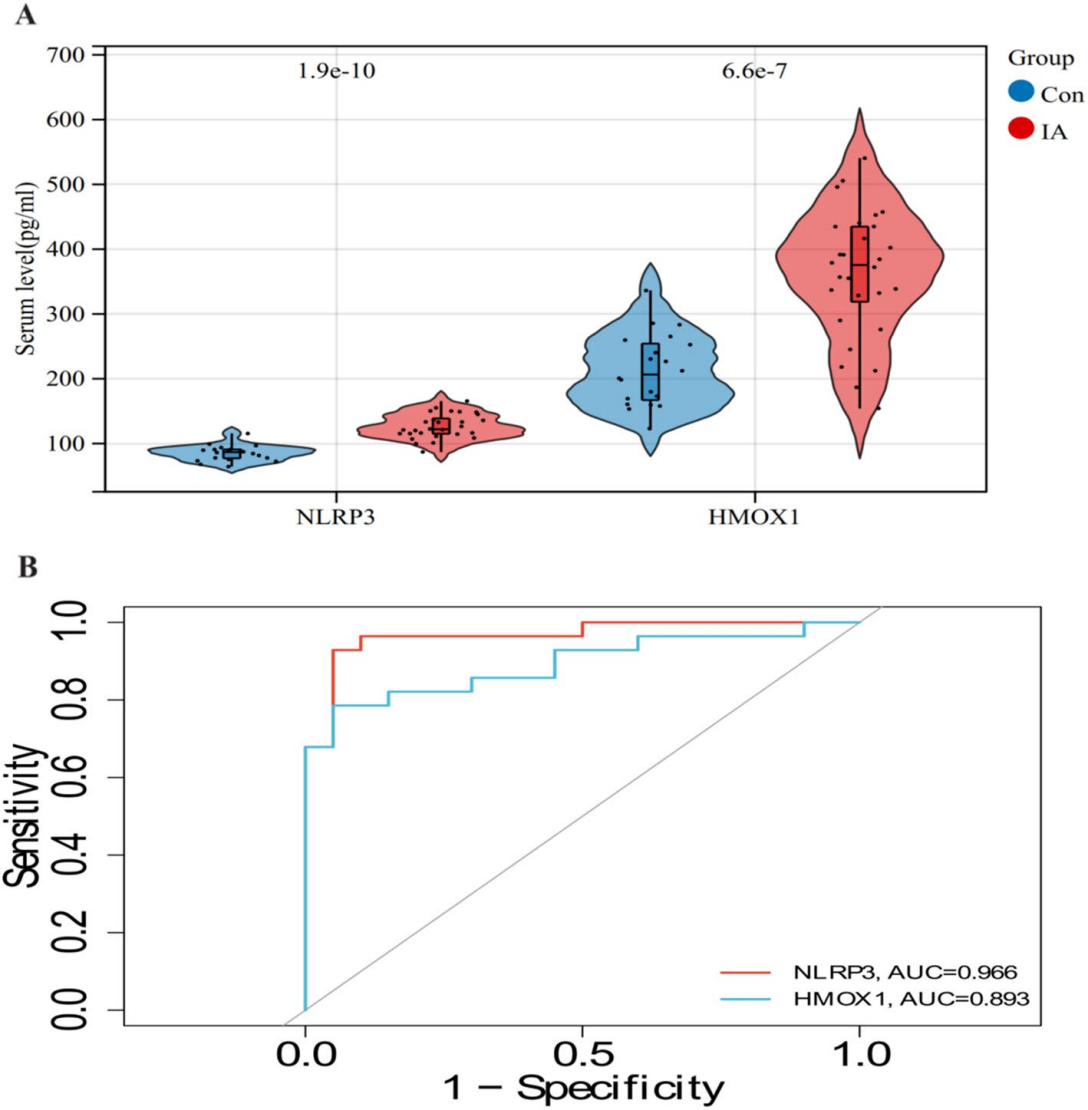
Validation of key genes in clinical samples. **A** Differences in key proteins between IA and normal groups (**B**) ROC-AUC distinguishes between IA and normal groups

In this study, the IA cohort consisted of 9 men and 19 women (age:54.25 ± 9.01). Among them, there were 7 cases of anterior communicating artery aneurysms, 6 cases of middle cerebral artery aneurysms, 9 cases of posterior communicating artery aneurysms, and 6 cases of basilar artery aneurysms, with a maximum diameter range of 5–15 mm. The control group consisted of 9 men and 11 women (age: 53.22 ± 7.20). Compared with the healthy group, serum levels of *NLRP3* (126.44 ± 18.94 vs 85.26 ± 18.90 pg/ml) and HO-1 (361.84 ± 97.79 vs 213.43 ± 55.43 pg/ml) were higher in the IA group (*P* < 0.001), consistenting with bioinformatics predictions (Fig. 7A). As shown in Fig. 7B, the ROC-AUC for distinguishing IA from normal group reached 0.966 for NLRP3 and 0.893 for HMOX1.

## Discussion

In this study, ferroptosis and ICD were found to play a role in the formation and rupture mechanisms of IA. Ferroptosis is an iron-dependent form of PCD, characterized by the accumulation of lipid peroxides. Its distinctive morphological features include mitochondrial atrophy, cristae loss, and increased membrane density. Its biochemical features encompass the intracellular accumulation of free iron, lipid reactive oxygen species (ROS), decreased cysteine uptake, and impaired glutathione (GSH) synthesis [14, 15]. Ferroptosis has been strongly associated with central nervous system disorders, such as cerebral hemorrhage, aging, and ischemic stroke. Elevated iron levels in neuronal cells after hemorrhagic stroke contribute to brain damage, whereas iron chelators attenuate this damage and exert neuroprotective effects [16]. Glutathione peroxidase 4 (GPX4)—a selenium-rich reductase—mitigates cellular damage caused by oxygen radicals by depleting two GSH molecules that interact with lipid peroxides [17].

In this study, HMOX1 upregulation was associated with IA. HMOX1 catalyzes the degradation of heme into biliverdin and Fe^2+^ ions in response to pro-oxidative and inflammatory stimuli, thereby contributing to ferroptosis [18]. HMOX1 expression increased significantly in primary mouse endothelial cells in a high-glucose/high-fat environment, correlating ferroptosis with diabetic atherosclerosis [19]. Additionally, excess heme levels elevated HMOX1 and Fe^2+^ in sickle cell mouse models [20]. After subarachnoid hemorrhage, ruptured erythrocytes release hemoglobin, which generates Fe^2+^ in conjunction with HMOX1. This phenomenon yields excessive free radicals via the Fenton reaction, promotes ROS generation, and exacerbates neuronal damage [21, 22]. Cerebral atherosclerosis is an independent risk factor for IA [23]. During cerebral atherosclerosis, erythrocyte leakage through neovascularization releases free hemoglobin, leading to vessel wall damage through HMOX1-mediated ferroptosis and facilitating the transition of vascular atherosclerosis to hemangioma [24]. A recent study on the formation of intracranial aneurysms (IA) has shown that silencing the KLF15 gene can upregulate HMOX1, promoting the occurrence of ferroptosis [25]. However, the specific regulatory mechanism of HMOX1 remains to be further investigated.

NLRP3 is a cytoplasmic multiprotein complex of the innate immune system that triggers pro-inflammatory pathways [26]. NLRP3 inflammasomes are involved in microglia-induced inflammatory responses and polarization [27, 28]. MitoQ reduces M1 marker expression and increases M2 marker expression both in vivo and in vitro after cerebral hemorrhage by inhibiting the ROS/NLRP3 inflammasome pathway [29]. Moreover, NLRP3 small interfering RNA shifts FeCl2-treated microglial polarization from M1 to M2 phenotype. In a stress-induced hypertension rat model, epinephrine produces ROS, triggering M1 phenotypic switching and NLRP3 activation. Additionally, MCC950—the NLRP3 inhibitor—reduces M1 polarization in the rostral ventrolateral medulla [30]. NLRP3 contributes to neuroinflammation and its inhibition ameliorates early brain injury and delayed cerebral vasospasm after subarachnoid hemorrhage. ROS activates NLRP3 inflammasomes, leading to caspase-1 activation. Activated caspase-1 proteolytically cleaves pro-inflammatory cytokines, amplifying the innate immune response and inducing pyroptosis. This cascade exacerbates neuronal cell death [31, 32]. Additionally, silent information regulator sirtuin 1 (SIRT1) inhibits NLRP3 inflammasome activation [33]. Saffron- and diosgenin-induced SIRT1 activation effectively inhibits NLRP3 inflammasome activity and attenuates neuroinflammation in mouse models of traumatic brain injury, protecting against subarachnoid hemorrhage [34, 35]. However, the specific mechanism by which SIRT1 regulates NLRP3 remains unclear and may involve ROS production.

## Limitations

This study presents several limitations that warrant attention. First, while NLRP3 and HMOX1 have been identified as key biomarkers, the lack of functional experiments prevents us from confirming their specific roles in IA pathogenesis. Functional studies are essential to validate their involvement in vascular remodeling, immune cell infiltration, and inflammatory processes. Future research, utilizing an IA mouse model, should aim to detect HMOX1 and ferroptosis markers such as ACSL4, SLC7A11, GPX4, and TFRC. In vitro experiments involving Human Umbilical Vein Endothelial Cells (HUVEC) exposed to H_2_O_2_ will simulate IA-related endothelial damage. By manipulating HMOX1 expression, we can further analyze its impact on ferroptosis. Secondly, the GEO dataset used in this study has a small sample size and is validated based on single-center data, which may reduce the generalizability of the research results and affect the robustness of the predictive model. Therefore, a larger multi-center dataset is needed. In addition, we will collect additional serum samples from patients with common neurological diseases such as stroke, arteriosclerosis, and Traumatic Brain Injury, detect the levels of NLRP3 and HMOX1, and compare them with those of IA patients to confirm their specificity.

## Conclusions

We identified two programmed cell death (PCD) genes, HMOX1 and NLRP3, which are differentially expressed and associated with intracranial aneurysms (IA), using in silico analysis. The validation of these results with serum samples from clinical studies enhances their reliability. Further research into these hub genes and their network-related genes is necessary for translating these findings into clinical applications for IA screening and therapy.

## Supplementary Information

Supplementary Material 1.

## Data Availability

This research analyzes the published data sets from Gene Expression Omnibus (http://www.ncbi.nlm.nih.gov/geo). The data used to support the findings of this study are available from the first author [Ning Gan], upon request.

## Abbreviations

aSAH: Aneurysmal subarachnoid hemorrhage
AUC: Area under curve
DEG: Differentially expressed genes
DT: Decision tree
eNet: ElasticNet
GEO: Gene Expression Omnibus
HMOX1: Heme Oxygenase 1
IA: Intracranial aneurysm
NLRP3: NOD-like receptor family pyrin domain containing 3
PCD: Programmed cell death
ROC: Receiver operating characteristic
SVM: Support vector machine
WGCNA: Weighted gene co-expression network analysis
Xgboost: EXtreme gradient boosting

## References

[1] Xu Z, Rui YN, Hagan JP, Kim DH. Intracranial aneurysms: pathology, genetics, and molecular mechanisms. Neuromolecular Med. 2019;21(4):325–43. 10.1007/s12017-019-08537-7. 31055715 10.1007/s12017-019-08537-7 PMC6829066

[2] Andreasen TH, Bartek J Jr, Andresen M, Springborg JB, Romner B. Modifiable risk factors for aneurysmal subarachnoid hemorrhage. Stroke. 2013;44(12):3607–12. 10.1161/STROKEAHA.113.001575. 24193807 10.1161/STROKEAHA.113.001575

[3] Løvik K, Laupsa-Borge J, Logallo N, Helland CA. Body composition and rupture risk of intracranial aneurysms. Eur J Med Res. 2024;29(1):297. 10.1186/s40001-024-01888-3. 38790007 10.1186/s40001-024-01888-3 PMC11127333

[4] Ishii D, Zanaty M, Roa JA, Li L, Lu Y, Allan L, et al. Postoperative cognitive dysfunction after endovascular treatments for unruptured intracranial aneurysms: a pilot study. Interv Neuroradiol. 2022;28(4):439–43. 10.1177/15910199211039917. 34516320 10.1177/15910199211039917 PMC9326860

[5] Fukuda M, Aoki T. Molecular basis for intracranial aneurysm formation. Acta Neurochir Suppl. 2015;120:13–5. 10.1007/978-3-319-04981-6_2. 25366592 10.1007/978-3-319-04981-6_2

[6] Moujalled D, Strasser A, Liddell JR. Molecular mechanisms of cell death in neurological diseases. Cell Death Differ. 2021;28(7):2029–44. 10.1038/s41418-021-00814-y. 34099897 10.1038/s41418-021-00814-y PMC8257776

[7] Zhou D, Zhu Y, Jiang P, Zhang T, Zhuang J, Li T, et al. Identifying pyroptosis- and inflammation-related genes in intracranial aneurysms based on bioinformatics analysis. Biol Res. 2023;56(1):50. 10.1186/s40659-023-00464-z. 37752552 10.1186/s40659-023-00464-z PMC10523789

[8] Zhu H, Tan J, Wang Z, Wu Z, Zhou W, Zhang Z, et al. Bioinformatics analysis constructs potential ferroptosis-related ceRNA network involved in the formation of intracranial aneurysm. Front Cell Neurosci. 2022;16:1016682. 10.3389/fncel.2022.1016682. 36313616 10.3389/fncel.2022.1016682 PMC9612944

[9] Wang S, Wang R, Hu D, Zhang C, Cao P, Huang J. Machine learning reveals diverse cell death patterns in lung adenocarcinoma prognosis and therapy. NPJ Precis Oncol. 2024;8(1):49. 10.1038/s41698-024-00538-5. 38409471 10.1038/s41698-024-00538-5 PMC10897292

[10] Hänzelmann S, Castelo R, Guinney J. GSVA: gene set variation analysis for microarray and RNA-seq data. BMC Bioinformatics. 2013;14:7. 10.1186/1471-2105-14-7. 23323831 10.1186/1471-2105-14-7 PMC3618321

[11] Riley RD, Ensor J, Snell KIE, Harrell FE Jr, Martin GP, Reitsma JB, et al. Calculating the sample size required for developing a clinical prediction model. BMJ. 2020;368:m441. 10.1136/bmj.m441. 32188600 10.1136/bmj.m441

[12] Zhou J, Ye Y, Chen Z, Liu Y, Wu B, Huang H. Upregulation of peripheral blood NLRP3 and IL-18 in patients with acute kidney injury in sepsis and its clinical significance. Immunity Inflamm Dis. 2024;12(12):e70113. 10.1002/iid3.70113. 10.1002/iid3.70113 PMC11653721 39692606

[13] Ulevicius J, Jasukaitiene A, Bartkeviciene A, Dambrauskas Z, Gulbinas A, Urboniene D, et al. Dysregulation of peripheral blood mononuclear cells and immune-related proteins during the early post-operative immune response in ovarian cancer patients. Cancers (Basel). 2023;16(1):190. 10.3390/cancers16010190. 38201617 10.3390/cancers16010190 PMC10778568

[14] Li J, Cao F, Yin HL, Huang ZJ, Lin ZT, Mao N, et al. Ferroptosis: past, present and future. Cell Death Dis. 2020;11(2):88. 10.1038/s41419-020-2298-2. 32015325 10.1038/s41419-020-2298-2 PMC6997353

[15] Liu J, Kang R, Tang D. Signaling pathways and defense mechanisms of ferroptosis. FEBS J. 2022;22:7038–50. 10.1111/febs.16059. 10.1111/febs.16059 34092035

[16] Pan F, Xu W, Ding J, Wang C. Elucidating the progress and impact of ferroptosis in hemorrhagic stroke. Front Cell Neurosci. 2023;16:1067570. 10.3389/fncel.2022.1067570. 36713782 10.3389/fncel.2022.1067570 PMC9874704

[17] Xie Y, Kang R, Klionsky DJ, Tang D. GPX4 in cell death, autophagy, and disease[J]. Autophagy. 2023;19(10):2621–38. 10.1080/15548627.2023.2218764. 37272058 10.1080/15548627.2023.2218764 PMC10472888

[18] Wu D, Hu Q, Wang Y, Jin M, Tao Z, Wan J. Identification of HMOX1 as a critical ferroptosis-related gene in atherosclerosis. Front Cardiovasc Med. 2022;9:833642. 10.3389/fcvm.2022.833642. 35498043 10.3389/fcvm.2022.833642 PMC9046663

[19] Meng Z, Liang H, Zhao J, Gao J, Liu C, Ma X, et al. HMOX1 upregulation promotes ferroptosis in diabetic atherosclerosis. Life Sci. 2021;284:119935. 10.1016/j.lfs.2021.119935. 34508760 10.1016/j.lfs.2021.119935

[20] Menon AV, Liu J, Tsai HP, Zeng L, Yang S, Asnani A, et al. Excess heme upregulates heme oxygenase 1 and promotes cardiac ferroptosis in mice with sickle cell disease. Blood. 2022;139(6):936–41. 10.1182/blood.2020008455. 34388243 10.1182/blood.2020008455 PMC8832481

[21] Deng X, Liang C, Qian L, Zhang Q. miR-24 targets HMOX1 to regulate inflammation and neurofunction in rats with cerebral vasospasm after subarachnoid hemorrhage. Am J Transl Res. 2021;13(3):1064–74. 33841640 PMC8014398

[22] Kang J, Tian S, Zhang L, Yang G. Ferroptosis in early brain injury after subarachnoid hemorrhage: review of literature. Chin Neurosurg J. 2024;10(1):6. 10.1186/s41016-024-00357-4. 38347652 10.1186/s41016-024-00357-4 PMC10863120

[23] Zhang Q, Liu H, Zhang M, Liu F, Liu T. Identification of co-expressed central genes and transcription factors in atherosclerosis-related intracranial aneurysm. Front Neurol. 2023;14:1055456. 10.3389/fneur.2023.1055456. 36937519 10.3389/fneur.2023.1055456 PMC10017537

[24] Galluzzi L, Vitale I, Aaronson SA, Abrams JM, Adam D, Agostinis P, et al. Molecular mechanisms of cell death: recommendations of the Nomenclature Committee on Cell Death 2018. Cell Death Differ. 2018;25(3):486–541. 10.1038/s41418-017-0012-4. 29362479 10.1038/s41418-017-0012-4 PMC5864239

[25] Fang G, Tian Y, You L, Xu R, Gao S. KLF15 prevents ferroptosis in vascular smooth muscle cells via interacting with p53. Biochem Biophys Res Commun. 2025;770:152029. 10.1016/j.bbrc.2025.152029. 40382847 10.1016/j.bbrc.2025.152029

[26] Shao BZ, Xu ZQ, Han BZ, Su DF, Liu C. NLRP3 inflammasome and its inhibitors: a review. Front Pharmacol. 2015;6:262. 10.3389/fphar.2015.00262. 26594174 10.3389/fphar.2015.00262 PMC4633676

[27] Zhang Y, Hou B, Liang P, Lu X, Wu Y, Zhang X, et al. TRPV1 channel mediates NLRP3 inflammasome-dependent neuroinflammation in microglia. Cell Death Dis. 2021;12(12):1159. 10.1038/s41419-021-04450-9. 34907173 10.1038/s41419-021-04450-9 PMC8671551

[28] Cai L, Gong Q, Qi L, Xu T, Suo Q, Li X, et al. ACT001 attenuates microglia-mediated neuroinflammation after traumatic brain injury via inhibiting AKT/NFκB/NLRP3 pathway. Cell Commun Signal. 2022;20(1):56. 10.1186/s12964-022-00862-y. 35461293 10.1186/s12964-022-00862-y PMC9035258

[29] Ma Q, Chen S, Hu Q, Feng H, Zhang JH, Tang J. NLRP3 inflammasome contributes to inflammation after intracerebral hemorrhage. Ann Neurol. 2014;75(2):209–19. 10.1002/ana.24070. 24273204 10.1002/ana.24070 PMC4386653

[30] Lei P, Li Z, Hua Q, Song P, Gao L, Zhou L, et al. Ursolic acid alleviates neuroinflammation after intracerebral hemorrhage by mediating microglial pyroptosis via the NF-κB/NLRP3/GSDMD pathway. Int J Mol Sci. 2023;24(19):14771. 10.3390/ijms241914771. 37834220 10.3390/ijms241914771 PMC10572659

[31] Jin J, Duan J, Du L, Xing W, Peng X, Zhao Q. Inflammation and immune cell abnormalities in intracranial aneurysm subarachnoid hemorrhage (SAH): relevant signaling pathways and therapeutic strategies. Front Immunol. 2022;13:1027756. 10.3389/fimmu.2022.1027756. 36505409 10.3389/fimmu.2022.1027756 PMC9727248

[32] Hu X, Yan J, Huang L, Araujo C, Peng J, Gao L, et al. INT-777 attenuates NLRP3-ASC inflammasome-mediated neuroinflammation via TGR5/cAMP/PKA signaling pathway after subarachnoid hemorrhage in rats. Brain Behav Immun. 2021;91:587–600. 10.1016/j.bbi.2020.09.016. 32961266 10.1016/j.bbi.2020.09.016 PMC7749833

[33] Chen H, Deng J, Gao H, Song Y, Zhang Y, Sun J, et al. Involvement of the SIRT1-NLRP3 pathway in the inflammatory response. Cell Commun Signal. 2023;21(1):185. 10.1186/s12964-023-01177-2. 37507744 10.1186/s12964-023-01177-2 PMC10375653

[34] Shaheen MJ, Bekdash AM, Itani HA, Borjac JM. Saffron extract attenuates neuroinflammation in rmTBI mouse model by suppressing NLRP3 inflammasome activation via SIRT1. PLoS ONE. 2021;16(9):e0257211. 10.1371/journal.pone.0257211. 34506597 10.1371/journal.pone.0257211 PMC8432768

[35] Zhang XS, Lu Y, Li W, Tao T, Wang WH, Gao S, et al. Cerebroprotection by dioscin after experimental subarachnoid haemorrhage via inhibiting NLRP3 inflammasome through SIRT1-dependent pathway. Br J Pharmacol. 2021;178(18):3648–66. 10.1111/bph.15507. 33904167 10.1111/bph.15507

